# Improved Method for Measuring the Permeability of Nanoporous Material and Its Application to Shale Matrix with Ultra-Low Permeability

**DOI:** 10.3390/ma12091567

**Published:** 2019-05-13

**Authors:** Taojie Lu, Ruina Xu, Bo Zhou, Yichuan Wang, Fuzhen Zhang, Peixue Jiang

**Affiliations:** 1 Department of Energy and Power Engineering, Tsinghua University, Beijing 100084, China; ltj17@mails.tsinghua.edu.cn (T.L.); ruinaxu@tsinghua.edu.cn (R.X.); zhou-b12@tsinghua.org.cn (B.Z.); wyc16@mails.tsinghua.edu.cn (Y.W.); zhangfuzhen@tsinghua.edu.cn (F.Z.); 2 Tsinghua University–University of Waterloo Joint Research Center for Micro/Nano Energy and Environment Technology, Beijing 100084, China; 3 Key Laboratory for Thermal Science and Power Engineering of Ministry of Education, Beijing 100084, China

**Keywords:** shale, permeability measurement, pressure decay method

## Abstract

Nanoporous materials have a wide range of applications in clean energy and environmental research. The permeability of nanoporous materials is low, which affects the fluid transport behavior inside the nanopores and thus also affects the performance of technologies based on such materials. For example, during the development of shale gas resources, the permeability of the shale matrix is normally lower than 10^−3^ mD and has an important influence on rock parameters. It is challenging to measure small pressure changes accurately under high pressure. Although the pressure decay method provides an effective means for the measurement of low permeability, most apparatuses and experiments have difficulty measuring permeability in high pressure conditions over 1.38 MPa. Here, we propose an improved experimental method for the measurement of low permeability. To overcome the challenge of measuring small changes in pressure at high pressure, a pressure difference sensor is used. By improving the constant temperature accuracy and reducing the helium leakage rate, we measure shale matrix permeabilities ranging from 0.05 to 2 nD at pore pressures of up to 8 MPa, with good repeatability and sample mass irrelevance. The results show that porosity, pore pressure, and moisture conditions influence the matrix permeability. The permeability of moist shale is lower than that of dry shale, since water blocks some of the nanopores.

## 1. Introduction

Porous materials are becoming increasingly popular in clean energy and environmental research, especially nanoscale porous media due to their large specific area. The permeability of nanoporous materials affects the fluid transport behavior inside the nanopores, and thus also affects the performance of technologies based on such materials [1,2]. 

In the last decade, shale gas has become more and more popular around the world due to its large reserves and cleanliness compared with traditional fossil fuels. The pore permeability characteristics of shale have an important influence on the determination of rock parameters, which are important to determine for the development of shale gas. The permeability represents the level to which a rock permits fluid flow at a given differential pressure. The common unit of permeability, the Darcy (D), equals 10^−12^ m^2^. The permeability of the rock determines the ability of shale gas to flow in the pore network, which in turn, affects the development value of the corresponding reservoirs.

Unlike conventional rock systems, the pore sizes of gas-bearing shales are almost at the nanometer scale. Pores smaller than 10 nm provide most of the specific surface area and pore volume for the storage of shale gas [3,4]. Loucks et al. observed various pores with diameters of between 5 and 750 nm in the Barnett Shale [5]. Furthermore, Zhou et al. observed a large number of pores with diameters of between 5 and 200 nm in organic matter of the shale of the Longmaxi formation [6]. In addition to nanopores, shale also has micro- and nanoscale natural fractures in clay or organic matter [7]. 

Moreover, shale formations contain non-negligible amounts of water. On the one hand, some shale reservoirs may be damaged by the process of geological water migration. Groundwater intrusion may cause high water saturation. On the other hand, shale gas exploitation often uses hydraulic fracturing. In the process of gas reservoir reconstruction, drilling fluid, completion fluid, cementing fluid, and fracturing fluid cannot be completely discharged from the rock, meaning that a significant portion of the water injected into a well will remain in the shale rock and other geological layers [8]. 

Different researchers have experimentally measured the permeability of dry shale in different shale blocks, including using plunger samples and particle samples [9,10,11,12,13]. Wang et al. summarized the shale permeability data of nearly eight blocks in North America [14]. The shale permeability obtained from various experimental studies using different measurement methods has a wide range from 0.01 nD to 1 mD. The shale permeability measured using core plug samples is several orders of magnitude higher than that obtained using fractured particle samples, due to the existence of large natural fractures in the plugs. The natural fractures contained in artificially fractured shale particles are much smaller than those in core plug samples, so the measured permeability of such particles is more reflective of the matrix permeability.

An increase in reservoir water saturation will lead to a decrease in gas permeability and an increase in the sensitivity of the reservoir to stress damage, which is unfavorable in the later stage of shale gas extraction [15]. In laboratory research, some researchers have observed a decrease in the permeability of shale plug samples after water saturation. For example, Shen et al. found that the permeability decreases with increasing water imbibition time [16]. Additionally, Ghanizadeh et al. observed that the measured permeability coefficients of a dry shale plug were significantly higher than those measured in the same plug with 1.1% water saturation [17]. 

Due to the limitations of micro-flow measurement technology, the steady-state permeability test method directly based on Darcy’s Law is difficult to apply in measurements of low-permeability and ultra-low-permeability materials for extremely long periods of time. For core samples with a permeability of 1 nD, the allowable air flow is only on the order of 10^−5^ cm^3^/s, even if a pressure difference of 10 MPa is applied on both sides, and such micro-flow is therefore hard to measure using the traditional steady-state method. In 1968, Brace et al. presented a low-permeability core-permeability measurement technology based on the pressure decay method. Compared with the steady-state method, the pressure decay method of permeability measurement greatly shortens the test time. In the decades following 1968, Hsieh et al. [18], Neuzil et al. [19], Dicker and Smits [20], Luffel et al. [21], Jones [22], Wu et al. [23], Cui et al. [24], and Barral et al. [25] continuously developed the theory and technology of this method. In 1993, Luffel et al. proposed a pressure decay measurement principle for low-permeability shale particles [21]. By applying an initial pressure difference to a particle, unsteady one-dimensional gas flow was made to occur from the outside to the inside along the radial direction of the particle.

However, when carrying out experiments on shale with a large pore pressure, it is difficult to measure relatively small pressure changes. For example, a widely used commercial shale matrix permeability measuring instrument has a maximum allowable pressure of 1.38 MPa (200 psi), and is strongly affected by the gas tightness of helium and temperature stability. Fisher et al. sent six samples of shale to leading service companies for permeability tests [26]. The results showed that differences in the measured permeability of up to four orders of magnitude were obtained by different laboratories when analyzing the same sample. Currently, measurements of shale permeability under moist conditions are usually obtained using plug samples. While it is known that water may be easily stored in large fractures, the permeability of moist shale matrix particles is still poorly understood due to a lack of study.

This study focuses on key issues regarding the pressure decay method for the measurement of the permeability of shale particle samples. To overcome the challenge of measuring small pressure changes at high pressure, a pressure difference sensor is used in the experimental system. By improving the constant temperature accuracy and reducing the helium leakage rate, we obtain the shale matrix permeability at pore pressures of up to 8 MPa with good repeatability and sample mass irrelevance. The results show that porosity, pore pressure, and moisture condition influence the matrix permeability. The permeability of moist shale is lower than that of dry samples, since water blocks some of the nanopores.

Using this improved method, a new shale-matrix permeability measuring instrument can be designed which has a higher allowable pressure and good repeatability. During shale reservoir assessment and shale gas exploitation, this method can provide a more effective means for the measurement of permeability, which is related to the development of shale gas resources.

## 2. Modeling and Methods

### 2.1. Pressure Decay Model

The core physical process of the experiment conducted in the present study is a dynamic process of pressure decay. Porous shale particles are kept at a constant temperature, and the test gas pressure in the pores is kept stable at the initial pore pressure *p*_1*i*_. Then, the pressure of the sample chamber is raised to *p*_0*i*_, which results in an imbalance between the free-space pressure and pore pressure of the particles and causes a radial flow from the surface to the internal space. Finally, the pressure reaches the steady-state pressure *p_f_*.

Unlike standard plunger samples, the size and geometry of the particles obtained by standard sieving are random variables subject to a certain statistical distribution. In order to establish a mathematical model to easily determine sample permeability, the particles are assumed to be spheres with a radius of *r*_0_. Under this assumption, the seepage problem degenerates into an unsteady one-dimensional radial flow. Gas flows along the radial direction (*r* direction) of a spherical particle.

In porous media, the mass conservation equation is as follows:

(1) $$\varepsilon \frac{\partial \rho }{\partial t}=-\frac{1}{r^{2}}\frac{\partial }{\partial r}(r^{2}\rho u)$$

where *ρ* is the gas density and *u* is the Darcy velocity of radial flow, ε is the porosity of porous media. Along with Darcy’s Law, the equation can be written as:

(2) $$\frac{\partial p}{\partial t}=\frac{1}{c_{g}\rho \varepsilon r^{2}}\frac{\partial }{\partial r}(\frac{r^{2}\rho K_{e}(p)}{\mu }\frac{\partial p}{\partial r})$$

where *µ* is the gas viscosity, *c_g_* is the isothermal compressibility, *K_e_* (*p*) is the apparent permeability, and *p* is the local pressure at a point inside the porous media.

The corresponding boundary conditions and initial conditions of the governing equations are:

(3) $${\left(\frac{\partial p}{\partial t}\right)}_{r=0,t}=0$$

(4) $$p(r<r_{0},t=0)=p_{1i},p(r=r_{0},t=0)=p_{0i}$$

(5) $$V_{f}{\left(\frac{\partial \rho }{\partial t}\right)}_{r=r_{0},t}=4\pi r_{0}{}^{2}{\left(-\frac{\rho K_{e}(p)}{\mu }\frac{\partial p}{\partial x}\right)}_{r=r_{0},t}$$

where *V_f_* is the volume of free space. Equation (5) describes how the gas in free space enters into the particle pore in the process of gas permeation.

Several researchers have provided solutions to such pressure decay equations. For instance, Profice et al. combined mass conservation with the Klinkenberg equations to give the solution [27]. Cui et al. developed an analytical method to calculate shale permeability [24]. They suggested that the slope of the formal stage of the logarithmic curve can be used to determine the permeability. Within the range of experimental pressure, the gas viscosity *µ* can be approximated as a constant. The test gas is assumed to be an ideal gas. Additionally, we define a non-dimensional number *f* which reflects the ratio of pore volume to cavity free-space volume:

(6) $$f=\frac{\varepsilon r_{0}(4\pi r_{0}{}^{2})}{V_{f}}=\frac{3V_{p}}{V_{f}}$$

where *V_p_* is the pore volume. Each volume satisfies the pressure equilibrium relation:

(7) $$p_{0i}V_{f}+p_{1i}V_{p}=p_{f}(V_{f}+V_{p})$$

**Figure 1:**
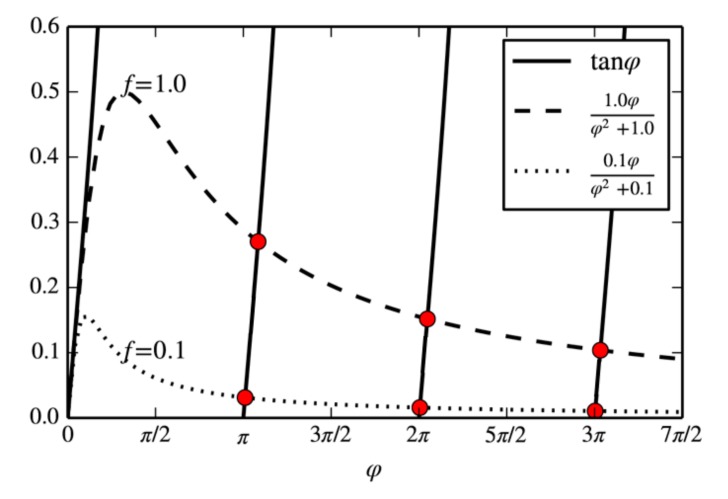
Graphical representation of the characteristic Equation (11). The solid lines represent the tangent function of *ϕ* and the dotted lines represent the fraction with different values of the non-dimensional number *f*. The red circles represent the characteristic roots of Equation (11). As *f* decreases, each characteristic root tends to be a positive integer multiple of π.

The apparent permeability *K_e_* (*p*) does not change during a single experiment as the pressure decay [24]. The dimensionless analytic solution of Equation (2) is:

(8) $$p(\tilde{r},\tilde{t})-p_{f}=(p_{0i}-p_{1i})\sum_{n=1}^{\infty }\frac{2\sin(\varphi_{n}\tilde{r})\exp(-\varphi_{n}{}^{2}\tilde{t})}{\tilde{r}[\varphi_{n}\cos\varphi_{n}+(2+f)\sin\varphi_{n}]}$$

where dimensionless radius and time are respectively:

(9) $$\tilde{r}=r/r_{0}$$

(10) $$\tilde{t}=t/t_{0},t_{0}=\frac{r_{0}{}^{2}\varepsilon \mu c_{g}}{K_{e}(\bar{p})},\bar{p}=\frac{1}{2}(p_{0i}+p_{1i})$$

and *ϕ_n_* are the characteristic roots of the following Equation (11). In one experiment, we define an average pressure to measure the permeability under this pressure, which is the mean value of the initial pore pressure *p*_1*i*_ and the sample chamber pressure *p*_0*i*_. As *f* decreases, each characteristic root tends to be a positive integer multiple of π, as shown in Figure 1.

(11) $$\tan\varphi =\frac{f\varphi }{\varphi^{2}+f}$$

**Figure 2:**
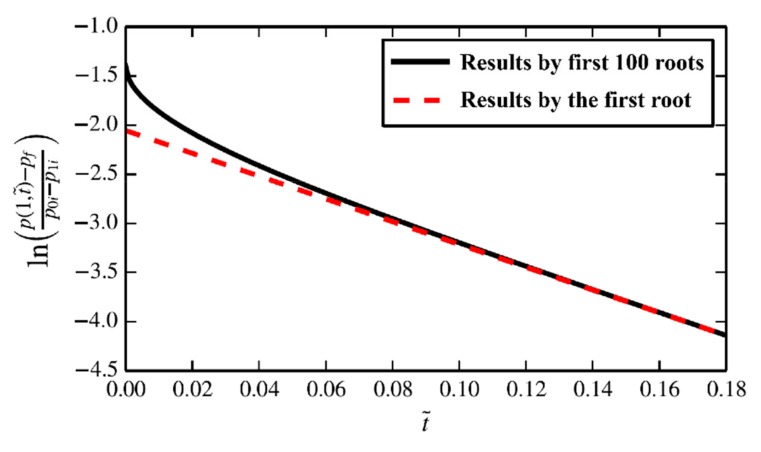
The truncation result of the analytic solution. The solid line represents the result of the truncation for the first 100 characteristic roots of Equation (11) and the dotted line represents the result of the truncation for the first characteristic root. When the dimensionless time is greater than 0.1, there is little difference between the two curves.

When the dimensionless time is greater than 0.1, the result calculated with the first characteristic root is almost consistent with the result obtained by taking the first 100 characteristic roots, as shown in Figure 2, which indicates that the first characteristic root dominates the whole pressure decay process. By solving the dimensionless equation of the pressure decay model, when the pressure decay time is over 0.1*t*_0_, we can use the slope of the logarithmic curve to calculate the permeability.

When only the first characteristic root is calculated, the function degenerates into an exponential function, and the fitting slope *S* of logarithmic pressure with time can be obtained:

(12) $$\left|S\right|=\frac{\varphi_{1}{}^{2}}{t_{0}}$$

(13) $$K_{e}=\frac{\left|S\right|r_{0}{}^{2}\varepsilon \mu c_{g}}{\varphi_{1}^{2}}=\frac{\left|S\right|r_{0}{}^{2}\varepsilon \mu }{\varphi_{1}^{2}}\frac{1}{\bar{p}}$$

### 2.2. Experimental Method

**Figure 3:**
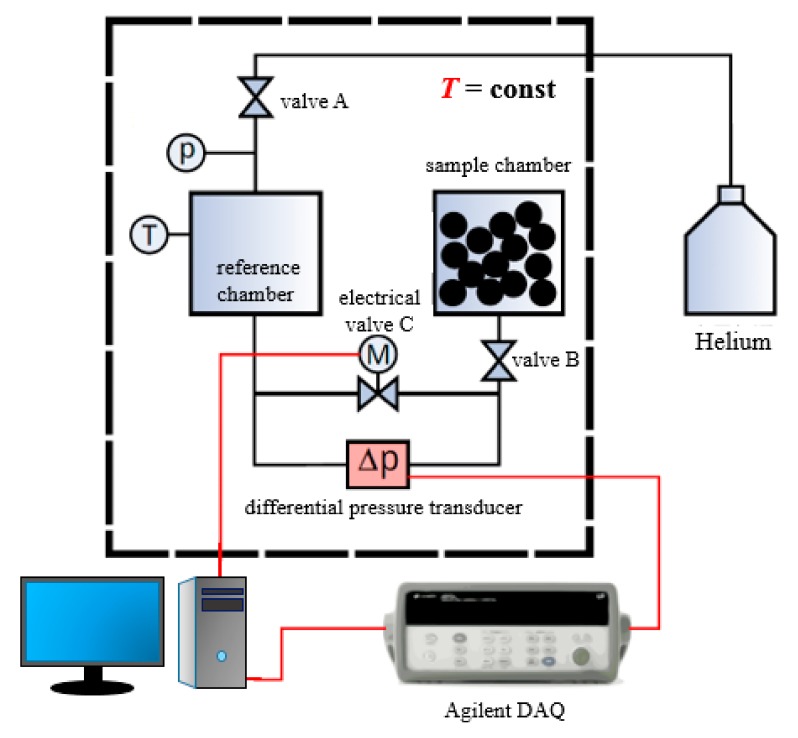
Schematic diagram of the experimental system used for the measurement of shale matrix particle permeability. Shale particle samples are placed in the sample chamber, and a reference chamber with approximately the same volume as the sample chamber is used for gas buffering. The electrical valve C is automatically controlled by the computer. The whole system is placed in a thermostatic water bath to maintain a constant temperature.

The experimental system for the measurement of shale matrix particle permeability is shown in Figure 3. The experimental system consists of a high-pressure helium source, a reference chamber, a sample chamber, a voltage regulator, a thermostatic water bath (accuracy ±0.05 °C), a pressure sensor, and other essential components which are connected by high-pressure sealing pipes. The helium cylinder is connected to an inlet pressure sensor (model EJA430A, Yokogawa Electric Corporation, Tokyo, Japan; range 0.14–14 MPa, accuracy 0.065%) via a pressure-reducing valve. An electric control valve is arranged on the pipeline connecting the reference chamber and the sample chamber. A high-pressure differential pressure transducer (model EJA110A, Yokogawa Electric Corporation; range 0.5–10 kPa, accuracy 0.075%) is set in parallel with the electric balance valve to record the pressure difference between the reference chamber and the sample chamber. A platinum resistance temperature sensor (accuracy ±0.1 °C) is placed in the reference chamber to record the temperature in the system. The entire core line is placed in the thermostatic water bath. To avoid current fluctuation, a voltage stabilizer is set along with the water bath.

**Figure 4:**
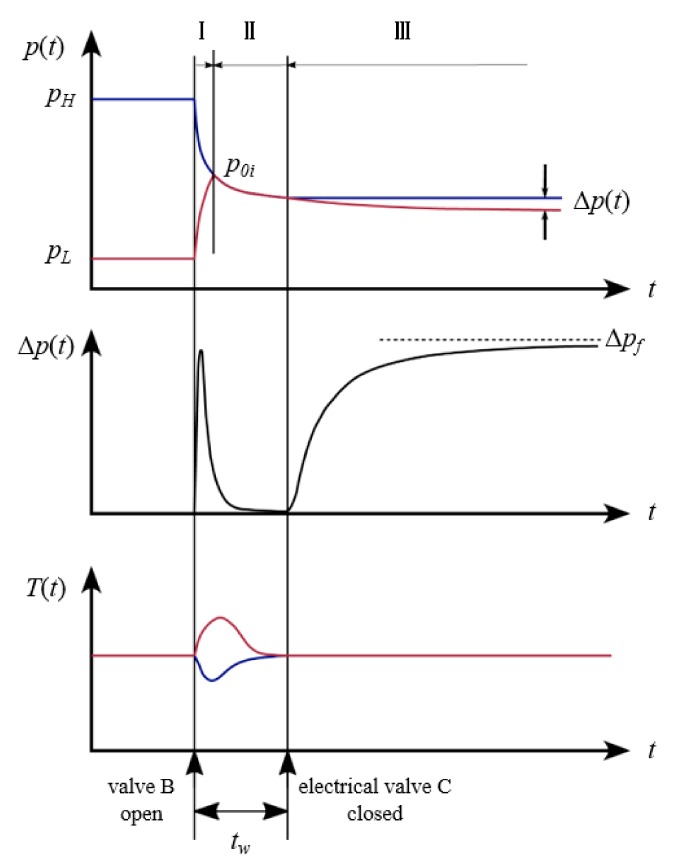
Graphs of the pressure *p*(*t*) of the reference chamber and sample chamber, the pressure difference signal Δ*p*(*t*) measured by the differential pressure transducer, and the gas temperature *T*(*t*) in the pipe.

Before the experiment, shale samples were crushed and passed through a standard sieve. A certain mass of particle samples was dried for over 12 h and then put into the sample chamber. The chamber volume is almost equivalent to that of the reference chamber. After securing the helium tightness of the device, the whole system was placed in the thermostatic water bath, and helium was pumped into the reference chamber at a certain pressure. After the system pressure had stabilized, we opened the sample chamber valve, causing high-pressure gas in the reference chamber to enter the low-pressure sample chamber. A pressure pulse is generated due to the valve action. When the whole free-space pressure is balanced at *p*_0*i*_, the electric control valve will automatically identify the pressure pulse and close the valve after a delay time *t_w_*. When the valve closes, the two chambers are separated; the pressure of the reference chamber is stable at *p*_0*i*__,_ while that of the sample chamber decays from *p*_0*i*_ to *p_f_*. The differential pressure sensor records the pressure difference between the two chambers. The diagram of pressure change is shown in Figure 4.

After the sample chamber valve is opened, the whole system will go through three main stages:

- Free-space pressure balance
  After the two chambers are connected, gas flows into the sample chamber from the reference chamber and the pipeline through the electric balance valve. When the pressure in the whole free space is basically balanced to *p*_0*i*_, the signal from the pressure difference transducer returns to zero. The duration of the entire free-space pressure balancing process depends on the pipe volume and flow resistance of the system.
- Free-space gas infiltration in both chambers
  As the gas gradually begins to infiltrate into the particle sample, the pressure of the free space drops. Until the electric balance valve is cut off, the output of the pressure difference sensor is always zero.
- Sample chamber free-space gas infiltration
  After the electric balance valve is cut off, only the gas in the sample chamber can infiltrate into the sample. The differential pressure curve of the two chambers, Δ*p*(*t*), is recorded by the differential pressure sensor. The differential pressure curve increases until the end of the pressure decay process, and the final output of the differential pressure sensor is denoted as Δ*p_f_*.

### 2.3. Experimental Materials

In this study, the aforementioned experimental system was used to measure the matrix permeability of four groups of shale samples from the Longmaxi formation.

**Figure A3:**
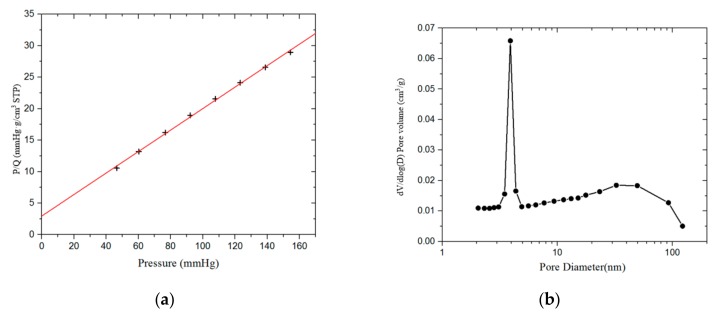
Nitrogen adsorption of sample 1 measured by an ASAP 2420 instrument (Micromeritics Instrument Corp., Norcross, GA, USA). (**a**) Langmuir surface area plot; (**b**) BJH desorption dV/dlog(D) pore volume.

**Figure A4:**
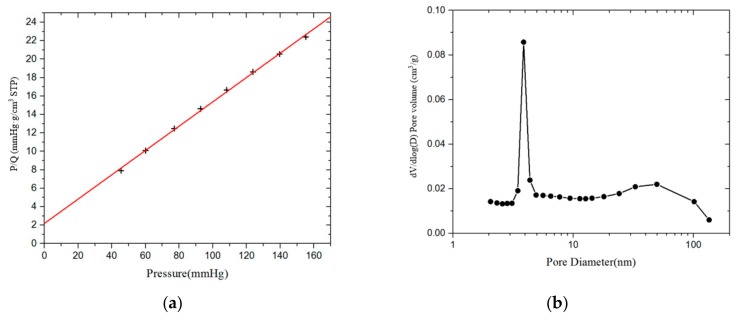
Nitrogen adsorption of sample 2 measured by an ASAP 2420 instrument (Micromeritics). (**a**) Langmuir surface area plot. (**b**) BJH desorption dV/dlog(D) pore volume.

**Figure A5:**
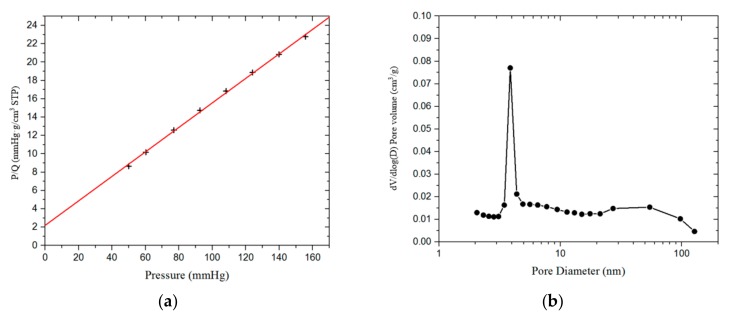
Nitrogen adsorption of sample 3 measured by an ASAP 2420 instrument (Micromeritics). (**a**) Langmuir surface area plot; (**b**) BJH desorption dV/dlog(D) pore volume.

**Figure A6:**
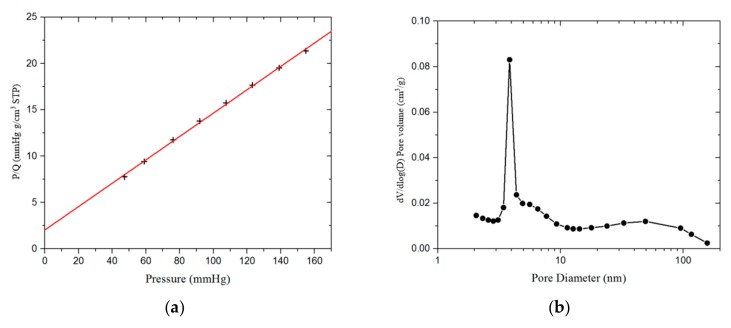
Nitrogen adsorption of sample 4 measured by an ASAP 2420 instrument (Micromeritics). (**a**) Langmuir surface area plot; (**b**) BJH desorption dV/dlog(D) pore volume.

Before the experiment, the petrophysical properties related to porosity and permeability were measured by the PetroChina Research Institute of Petroleum Exploration and Development in Langfang. The results are summarized in Table 1 and Table 2. TOC means the Total Organic Carbon. More data and figures can be seen in Appendix A Figure A3, Figure A4, Figure A5 and Figure A6.

### 2.4. Data Processing

The sample particles were passed through sieves with different mesh sizes. Each experiment was kept under average pressure for more than 2000 s. The constant temperature was set at 30 °C. For the experiment, 20–35 mesh shale particles with a mass of 10 g were chosen. The initial pressure was maintained at around 1 MPa and the average pressure changes were kept within the range of 1–8 MPa.

By calculating the pressure decay curve logarithmically, the pressure decay process goes into the formal stage after the initial time. Using the result of the pressure decay model equation, the permeability under different pressures can be calculated using the slope fitted for the linear stage of the logarithmic curve. An example of the data processing of sample 1 is described in Appendix A.

## 3. Results

### 3.1. Repeatability of Experiment and Mass Influence

**Figure 5:**
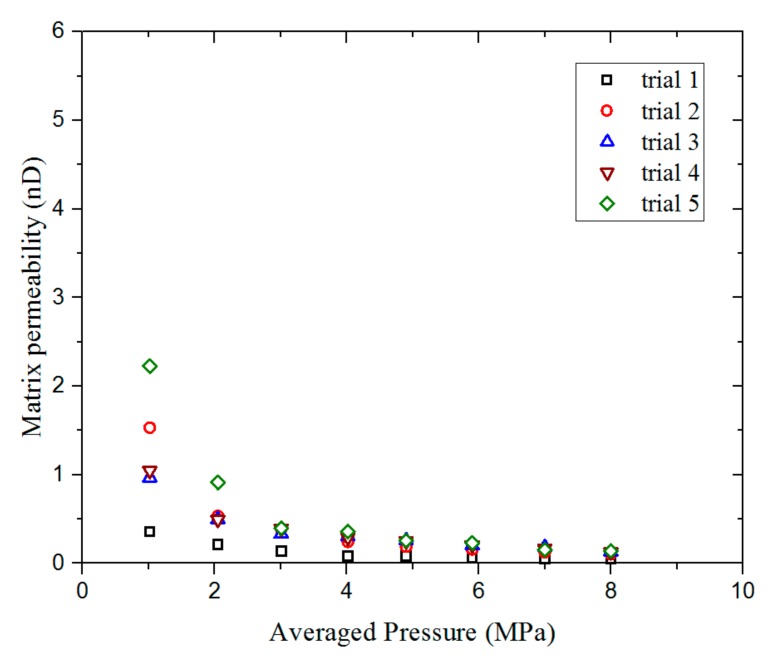
The results of the repeatability experiment, involving five trials, using sample 1 under pressures of 1–8 MPa.

Firstly, we used sample 1 to determine the repeatability of the experiment. For the experiment, 20–35 mesh shale particles with a mass of 10 g were chosen. The initial pressure difference was maintained at around 1 MPa and the average pressure changes were kept within the range of 1–8 MPa. Figure 5 shows the permeability values obtained from five trials using sample 1. The difference in the matrix permeability determined in the five experiments was smaller than 1 nD. When the average pore pressure increases, this difference gradually reduces; at a pore pressure of 8 MPa, the difference is almost within 0.1 nD. The results of the repeatability experiments demonstrate the stability of the permeability measurements obtained using the experimental system. 

**Figure 6:**
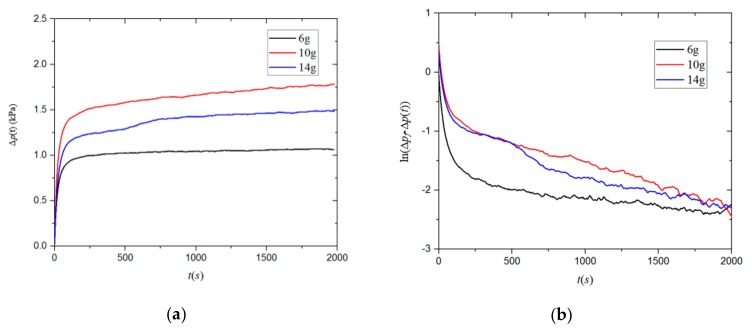
(**a**) The pressure difference signal Δp(*t*) and (**b**) logarithmic pressure decay curve for sample 1 measured at a pressure of 3 MPa and a temperature of 30 °C The black, red, and blue lines indicate sample masses of 6, 10, and 14 g, respectively.

Secondly, we investigated the effect of sample quality on the shale matrix permeability. For the four groups of samples, 6, 10, and 14 g of 20–35 mesh particle samples were respectively put in the sample chamber, and experiments were carried out under a pressure of 3 MPa and a temperature of 30 °C. Figure 6 shows the pressure difference signal and logarithmic pressure decay curve for sample 1.

**Figure 7:**
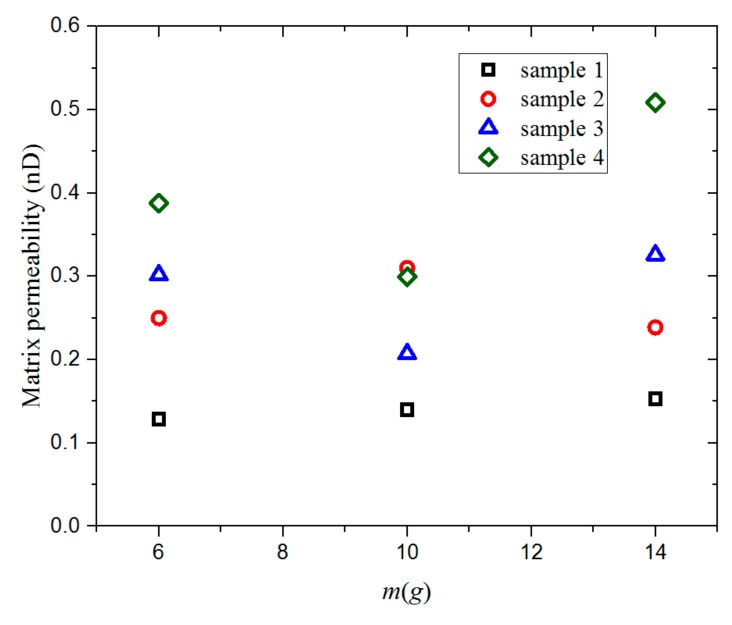
The permeability values of four groups of shale samples for sample masses of 6, 10, and 14 g measured under a pressure of 3 MPa and a temperature of 30 °C.

Figure 7 shows the permeability values of the four groups of samples for different sample masses. For the same sample group, the permeability differs by less than 0.2 nD for different sample masses under the same experimental conditions. The results show that the matrix particle permeability is not sensitive to the change of sample mass. 

### 3.2. The Effects of Pressure on Permeability

**Figure 8:**
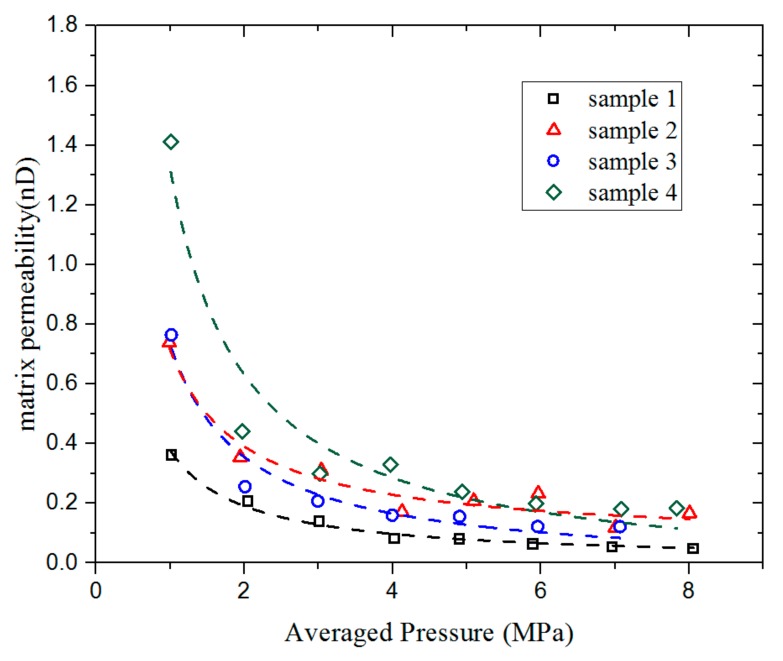
Matrix permeability of four shale particle samples under different pressures. The dotted line is the result of the fitting by the Klinkenberg equation.

To investigate the effect of pressure on the estimated permeability, we chose a total of 20–35 mesh shale particles with a mass of 10 g. The initial pressure difference was maintained at around 1 MPa and the average pressure changes were kept within the range of 1–8 MPa. Figure 8 shows the permeability values of the four groups of samples under different pressure conditions. It can be seen from the figure that the overall permeability of the shale matrix particles decreases with increasing pressure, especially at low pressure; this corresponds to the Klinkenberg effect. 

**Figure 9:**
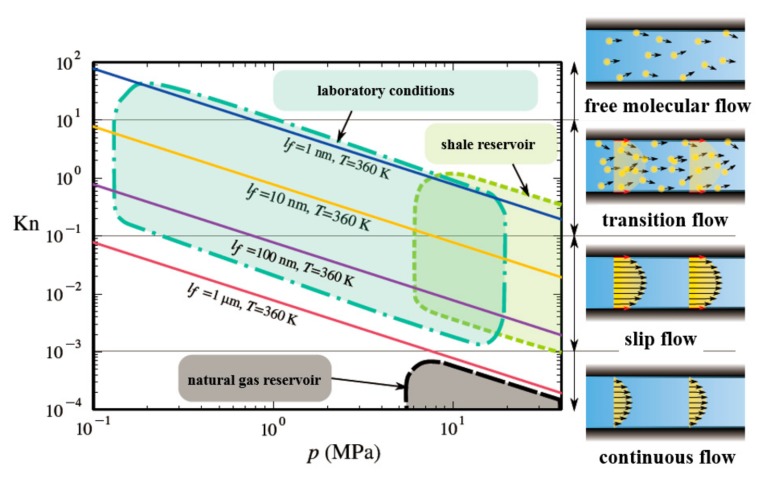
Rarefied gas flow type under different conditions. This can be divided into free molecular flow (*K_n_* > 10), transition flow (0.1 < *K_n_* < 10), slip flow (10^−3^ < *K_n_* < 0.1), and continuous flow (*K_n_* < 10^−3^).

The fundamental reason why the permeability of shale matrix is different from that of conventional reservoir rocks is the nanoscale characteristics of fluid flow. Under the temperature and pressure of shale reservoirs, the flow of methane gas in the nanopores is a rarefied gas flow, as shown in Figure 9. The Knudsen number describes the ratio of the mean free path of a gas molecule *λ* and the characteristic flow length *l_f_*:

(14) $$K_{n}=\frac{\lambda }{l_{f}}$$

In the nanoscale pores of shale, the flow state of gas is mainly slip flow and transition flow. In the slip zone, the gas retains the characteristics of the continuous medium, however has a non-zero velocity on the solid boundary.

In 1945, Klinkenberg studied the slip flow of low-permeability rock caused by the gas rarefaction effect, and found that the rock permeability measured by liquid medium was a constant independent of pressure, while the permeability measured by gaseous medium increased with decreasing experimental pressure. Klinkenberg concluded that the slip velocity of gas on the rock pore surface caused the correlation between permeability and pressure. He derived the following Klinkenberg equation based on a laminar flow model considering slip velocity in a one-dimensional uniform pipe [28]:

(15) $$K_{e}=K_{\infty }(1+\frac{b_{k}}{\bar{p}})$$

where *K_e_* is the apparent permeability of the rock, *K_∞_* is the absolute permeability of the rock, and *b_k_* is the Klinkenberg slip constant. Different researchers have carried out experimental fitting and theoretical derivation for the slip constant in Equation (15) [9,29,30,31].

The fluid property in confined nanopores is different from the large-scale fluid property, due to the influence of capillarity, interfacial phenomena, and hydrodynamics. For regularly shaped structures such as hard-sphere nanopores or nanochannels, theoretical and simulation methods, such as density functional theory and Monte Carlo simulation, are effective ways to describe the fluid mechanics [32,33,34].

In our study, since the pressure ranges from 1 to 8 MPa, the flowing gas is in the transition zone and slip zone. The gas maintains the properties of a continuous medium with a velocity at the boundary of the solid, so the basic equations are still used. Further research is warranted into the apparent permeability of shale matrix, the absolute permeability model, and the more acute fluid property in confined nanopores.

### 3.3. Permeability of Moist Shale Particles

The moist permeability was measured for shale particle sample 1 used in the previous dry sample experiment. After a long period of vacuum treatment, we added water to the vessel and left it for over 48 h to allow saturation. Water on the surface of the rock was then removed by filter paper. By the weighing method, the water saturation rate was determined to be 1.5%. 

To analyze the differences between the two samples, the particles between the dry and moist samples were scanned with a Nuclear Magnetic Resonance (NMR) instrument. Low-field NMR is a non-destructive technique for the quantitative measurement of hydrogen-containing fluids in porous media. Here, we use the T_2_ time to reflect different transverse relaxation times of hydrogen-containing media of different scales in pores. A shorter T_2_ time corresponds to a smaller amount of fluid. Researchers have demonstrated that a T_2_ of around 1 ms contains a signal from nanopores in kerogen [35,36,37].

**Figure 10:**
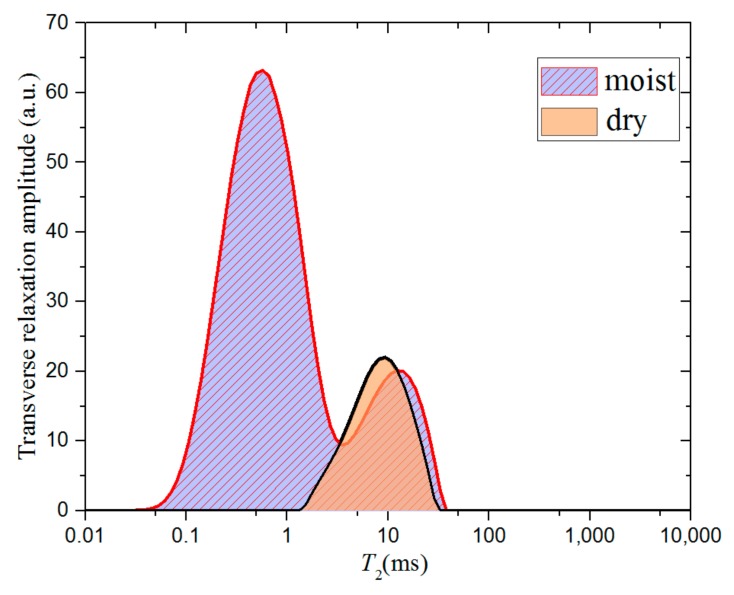
Transverse relaxation amplitude of shale particle sample 1 detected by low-field Nuclear Magnetic Resonance. The *T*_2_ measuring sequence is CPMG.

The change in the transverse relaxation amplitude after water saturation is shown in Figure 10. For dry samples, we consider that after a long drying time, the remaining shale gas in the pores had almost all escaped, and the signal comes from the solid skeleton of a rock sample. In NMR signal the peak of relaxation amplitude appears between T_2_ values of 0.1–1 ms, which we believe represents the water molecule in the nanopores of the shale rock.

**Figure 11:**
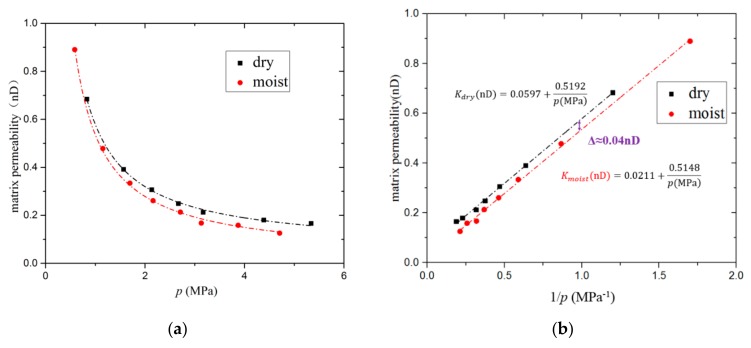
Matrix permeability of dry and moist (1.5% water) samples of shale sample 1 under different pressures. (**a**) Matrix permeability vs. pressure. The dotted lines are the results of fitting using the Klinkenberg equation. (**b**) Matrix permeability vs. 1/*p*.

The permeability of moist samples were measured in a similar way to dry samples. The permeability of the same sample decreases after water saturation under the same pressure, as shown in Figure 11. The two permeability vs. pressure curves (i.e., for dry and moist samples) were fitted using the Klinkenberg equation. It can be seen from the fitting relations that the absolute permeability of the dry and moist samples differs by about 0.04 nD. According to the fitting relation, with increasing pressure, the influence of the moisture content on the matrix permeability gradually increases, with a maximum decrease in permeability of 30% being observed.

## 4. Discussion

When using the pressure decay method to measure the permeability of crushed samples, many researchers have extensively used commercial equipment. Achang et al. found that temperature regulation was the key to obtaining consistent results [38]. If the temperature fluctuates, the available data from the pressure decay curve is limited. This requires the experimental system to work steadily under constant temperature for a certain period of time. An air thermostat is usually used to maintain the temperature of the sample chamber. Air thermostats have the advantages of convenience and the fact that they have less impact on the design of the system. However, due to the smaller specific heat of air compared with water, the constant temperature measurement of an air thermostat may be slightly more unstable than that obtained using a similarly priced water bath. In our experiment, the temperature was maintained to within ±0.05 K.

Although the pressure decay method provides an effective way to measure low permeability, commercial apparatuses have difficulty measuring low permeability at high pressure conditions over 1.38 MPa. Therefore, Klinkenberg corrections and in situ effective stress corrections are essential to relate the results to real reservoirs [27,39,40,41]. Some laboratories run pressure decay tests at different pressures. However, the measured pressure curve consists of a number of discrete steps (e.g., Heller et al. [42]), which leads to difficulties in data processing. This is due to the fact that, when using a system based on the Luffel system, in which one pressure meter is used to record the whole system pressure, the total variation of the pressure decay curve is very small at a high pressure range, and is almost equal to the resolution of the pressure measurement. If one tries to use a system based on the Luffel system to measure the permeability of low-permeability rocks under a pressure of 10 MPa, the pressure change in the sample chamber will be quite small, and so the measured pressure curve will consist of discrete steps. Therefore, in this study, we attempted to use a pressure difference sensor that resists high pressure to measure the pressure change in the sample chamber. Since the pressure range of the differential pressure sensor is almost the same order of magnitude as the variation of the pressure decay curve, the measurement accuracy of the experimental system will be improved, and the measurement accuracy does not change with increasing system pressure within the pressure limit.

In pressure decay experiments on low-permeability particles, helium is often used as a test gas to reduce the effect of adsorption. The molecular weight of helium is very low, and tight sealing conditions are therefore required. Some researchers conducted a leak test for a commercial permeability-measuring apparatus, and recorded a leakage rate of <0.062 psi/s [24]. In our experimental system, a better sealing system is designed for the sample chamber; even at a pressure of 8 MPa, the leakage rate is lower than 0.025 kPa/min, which ensures the accuracy of the pressure measurement in the experiment.

In our study, matrix permeability showed little reduction after water saturation. However, natural shale formations have a multi-scale pore structure. In such formations, large cracks tend to be blocked easily, which is the main factor that leads to the reduction of permeability. Especially in low-permeability and ultra-low-permeability reservoirs, the pore throats, which allow the free flow of fluid, are small, and the capillary pressure is therefore large, which makes it more difficult to discharge water. Additionally, the spherical bubbles in oil or oil droplets in water need to overcome additional resistance due to interfacial tension during oil displacement. The water blocking effect and Jamin effect are disadvantageous to the exploitation of oil and gas resources. The damage index varies with the conditions of the formation. To better analyze the impact of moisture on permeability, a multi-parameter porosity and permeability model is needed [43,44].

In this study, permeability information for the shale matrix samples was calculated by analyzing the experimentally derived pressure decay curve. In the data processing, errors come from the accuracy of the model and the measurement of parameters.

**Figure 12:**
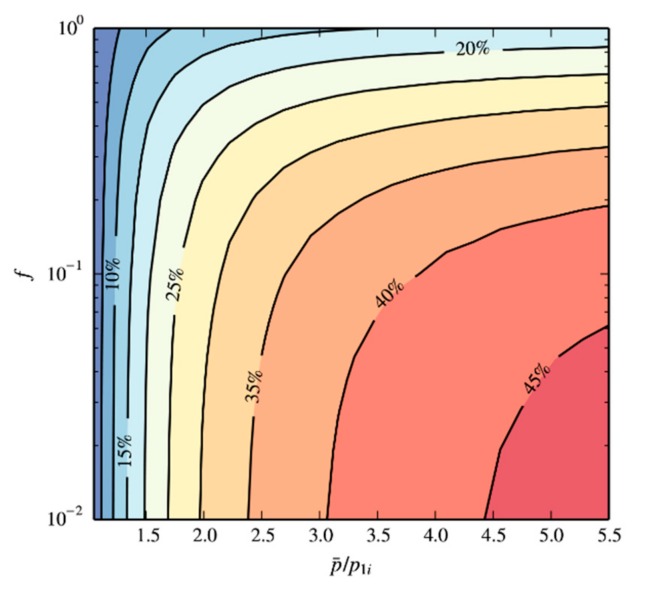
The relative error in the calculated permeability due to ignoring gas compressibility. For systems with a smaller dimensionless number *f*, when the system initial pressure ratio is large, ignoring the gas compressibility leads to a larger error in the calculated permeability.

When ignoring the effect of adsorption in the mathematical model, physical effects such as gas compressibility will affect the calculation of the apparent permeability. Here, we use a numerical method based on a finite-difference scheme to calculate the relative errors at different permeabilities due to ignoring compressibility, which are shown in Figure 12. With increasing initial pressure ratio ${\bar{p}/p}_{1i}$, the error due to ignoring compressibility increases. On the other hand, increasing the value of *f* can reduce the error under certain pressure conditions. This is due to the fact that, when the value of *f* is smaller, the first characteristic value *ϕ*_1_ is closer to π. Therefore, the pressure inside the particle will approach the final pressure *p_f_* more quickly, and *p_f_* will be close to *p*_0*i*_. In summary, for systems with a smaller dimensionless number *f*, when the system initial pressure ratio ${\bar{p}/p}_{1i}$ is large, ignoring the gas compressibility will lead to a larger error in the calculated permeability.

Additionally, the apparent permeability in low-permeability porous media is related to pressure. Assuming that the apparent permeability *K_e_* (*p*) does not change in a single experiment as the pressure decays leads to an error from ignoring the rarefaction effect. Considering the dimensionless equation:

(16) $$\frac{\partial p}{\partial \tilde{t}}=\frac{1}{\mu }\frac{1}{{\tilde{r}}^{2}}\frac{\partial }{\partial \tilde{r}}({\tilde{r}}^{2}\frac{K_{e}(p)}{K_{e}(\bar{p})}\frac{p}{\bar{p}}\frac{\partial p}{\partial \tilde{r}})$$

where the permeability radio $K_{e}(p)/K_{e}(\bar{p})$ has the same status with pressure ratio $p/\bar{p}$ in the approximate solution. Thus, similar to the error analysis for gas compressibility, for systems with smaller dimensionless number *f*, when the permeability ratio $K_{e}(\bar{p})/K_{e}(p_{1i})$ is large, ignoring the rarefaction effect of gas will lead to a larger error in permeability. 

In our study, we mainly use shale particles of around 20 mesh, the diameter of which is around 1 mm. Larger particles may not meet the assumption of sphericity, so that the one-dimensional model cannot be applied. When the particle size continues to increase, there may be a small number of macropores in the particle, which changes the permeability of the particles. Cui et al. [24] used a dual-porosity model which takes into account the changes in particle permeability caused by micropores and macropores, and obtained an empirical formula for the permeability. The equivalent permeability (*K_e_*) determined using the methods proposed in the present study should be related to the permeability of macropores (*K_a_*) and micropores (*K_i_*) as follows:

(17) $$\frac{1}{K_{e}}=\frac{1}{K_{a}}+\frac{C_{0}R_{i}{}^{2}}{R_{a}{}^{2}K_{i}}$$

where *C*_0_ is a constant related to the size of the micropores in the particles (*R_i_*), rock particle size (*R_a_*), macroporosity, and microporosity. 

**Figure 13:**
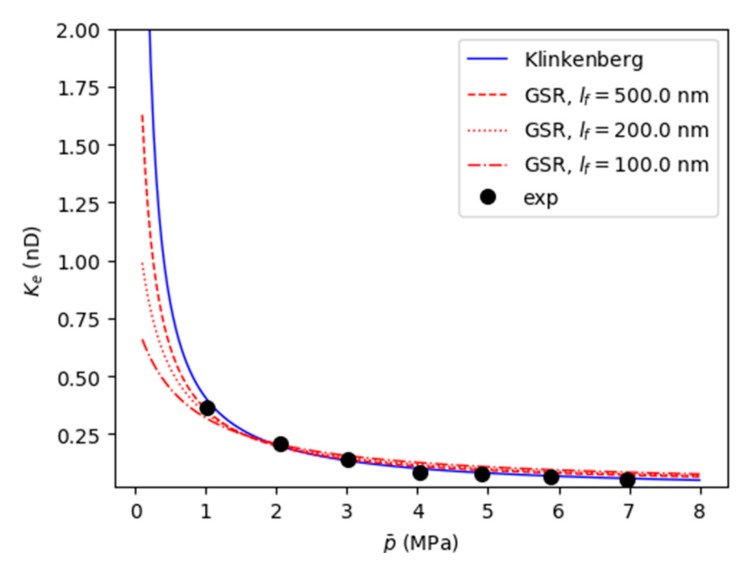
A comparison of the permeability fitting using the general slip regime (GSR) model and the Klinkenberg model. The characteristic flow scales *l_f_* used for the fitting with the GSR model were 100, 200, and 500 nm.

The Klinkenberg model describes a theoretical permeability model as a function of the gas pressure. However, the Klinkenberg relation was derived based on the formula of the mass flow rate in cylindrical tubes [28]. It can be seen from the formula that the permeability will increase to infinity at low pressure, which is not physically possible. The reason for this failure is the assumption that the porous medium is a bundle of tubes with uniform cross-sections. Zhou et al. improved the Klinkenberg permeability model for porous media with pore-scale wall-slip considering pore geometry complexity [45], creating an effective permeability model, called the general slip regime (GSR) model. The model can be expressed as follows:

(18) $$K_{e}=K_{\infty }(\frac{1+S_{1}\sigma K_{n}}{1+S_{2}\sigma K_{n}})$$

where *S*_1_ and *S*_2_ are REV geometry dependent rank 2 tensors, and *K_∞_* is the liquid permeability which does not involve the wall-slip effect for gas flow. When Kn < 0.01, the GSR model can be linearized to the Klinkenberg model. When Kn ≤ 0.1, the Klinkenberg model of apparent permeability is overvalued by 15–70%. Here, we use the GSR model to fit the permeability results by pressure for comparison with the Klinkenberg model, as shown in Figure 13. At low pressure, the permeability obtained using the GSR model is lower than that obtained using the Klinkenberg model. When the characteristic flow length is small, the Knudsen number is larger and the difference in the permeabilities obtained by the two models is more pronounced.

## 5. Conclusions

An experimental investigation of the permeability of shale matrix was conducted using the pressure decay method. The conclusions of this investigation are as follows:

1. The proposed experimental method for the measurement of low permeability represents an improvement over previous methods. To overcome the challenge of measuring small pressure change at high pressure, a pressure difference sensor is used. By improving the constant temperature accuracy and reducing the leakage rate of helium, we obtain the shale matrix permeability at pressures of up to 8 MPa and pore pressures ranging from 0.05 to 2 nD, with good repeatability and sample mass irrelevance.
2. As gas molecules inside nanopores are affected by the Klinkenberg slip effect, the apparent permeability is larger when measured at low pressure. With increasing pressure, the permeability measured under high pressure is closer to the absolute permeability of the particles.
3. The permeability of moist shale is lower than that of dry shale, since water blocks some of the nanopores. In natural shale formations, large cracks tend to fill with water more easily, which leads to the reduction of permeability.

## Figures and Tables

**Table 1:** Petrophysical properties of the shale particle samples.

Serial Number	Depth (m)	TOC (%)	Sulfur (%)	Porosity (%)
1	2321.66	2.1	2.5	2.14
2	2329.76	3.9	4.8	2.90
3	2338.84	4.1	6.3	3.62
4	2346.01	5.9	4.2	4.54

**Table 2:** Specific surface area and pore size distribution of the shale particle samples.

Serial Number	Specific Surface Area ^1^ (m^2^/g)	Cumulative Pore Volume ^2^ (cm^3^/g)	Average Pore Diameter A ^1^ (nm)	Average Pore Diameter B ^2^ (nm)
1	18.4309	0.028707	6.55	9.32
2	23.9007	0.035465	6.33	9.26
3	23.4238	0.028691	5.47	8.28
4	24.6843	0.026502	4.80	7.25

^1^ Data processed with the BET method. ^2^ Data processed with the BJH method.

**Figure A1:**
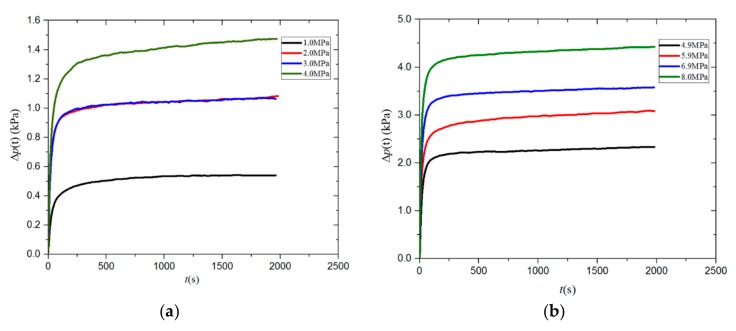
Pressure difference signal (Δp(*t*)) for shale particle sample 1 measured by the differential pressure transducer under pressures of 1–8 MPa. (**a**) average pore pressure under 1–4 MPa with smaller Δp*_f_*; (**b**) average pore pressure under 5–8 MPa with larger Δp*_f_*.

**Figure A2:**
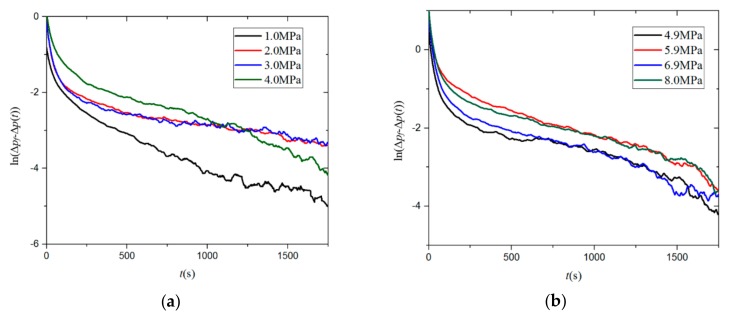
Logarithmic pressure decay curve, ln(Δp*_f_*-Δp(*t*)), for shale particle sample 1 under pressures of 1–8 MPa. (**a**) average pore pressure under 1–4 MPa; (**b**) average pore pressure under 5–8 MPa.

## Appendix A

Here, we take sample 1 as an example to describe the data processing procedure. Figure A1 shows the pressure difference curve detected by the pressure difference sensor under different equilibrium pressures.

After the pressure pulse, the pressure inside the sample chamber decreases continuously, and the pressure difference between the sample chamber and the reference chamber gradually increases. By displaying the pressure decay curve logarithmically, as shown in Figure A2, it can be seen that at the early stage of the pressure decay process, the free-space gas rapidly enters the pores of the matrix particles, and the curve drops sharply. Then, the gas infiltration and migration slows down, and the curve tends to be linear. According to the dimensionless result, when the dimensionless time is greater than 0.1, the pressure decay process goes into the formal stage. Using Equation (13), we can calculate the sample permeability under different pressures using the slope fitted at the linear stage of the logarithmic curve.
